# MCP‐1 Is Elevated in the Cerebral Fluid of Children With Tourette Syndrome: Case Series and Literature Review

**DOI:** 10.1002/brb3.70617

**Published:** 2025-06-10

**Authors:** Ke Zhongling, Chen Mengxin, Huang Yuxian, Chen Yanhui

**Affiliations:** ^1^ Department of Pediatrics Fujian Medical University Union Hospital Fuzhou China

**Keywords:** CSF, immunologic, MCP‐1, neuroinflammation, Tourette syndrome

## Abstract

**Purpose:**

This study aims to investigate cerebrospinal fluid (CSF) in patients with Tourette syndrome (TS) to identify the role of neuroinflammation in the pathophysiology of TS.

**Methods:**

We retrospectively reported cerebrospinal fluid (CSF) examination in four male adolescents diagnosed with severe TS, as indicated by a high Yale Global Tic Severity Scale (YGTSS) score. The examination included routine and biochemical tests, oligoclonal band testing, and analysis of 14 neural autoantibodies. Furthermore, the levels of 34 cytokines were also measured.

**Findings:**

CSF examinations revealed that routine and biochemical tests were normal and that no oligoclonal bands were detected. The 14 neural autoantibodies tested were negative. Among the 34 cytokines analyzed, only monocyte chemoattractant protein‐1 (MCP‐1) levels were significantly elevated.

**Conclusions:**

This study is the first to report elevated levels of MCP‐1 in the CSF of patients with TS. Our findings suggest that MCP‐1‐associated neuroinflammation may play a crucial role in the pathogenesis of TS, indicating that targeting MCP‐1 could be a promising therapeutic approach to managing symptoms of TS.

## Graphical abstract

Our findings suggest that MCP‐1‐associated neuroinflammation may play a crucial role in the pathogenesis of TS, indicating that targeting MCP‐1 could be a promising therapeutic approach for managing TS symptoms.

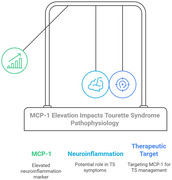

## 1 Background

Tourette syndrome (TS) is a common neurodevelopmental disorder in children characterized by one or more involuntary motor tics and at least one vocal tic that persists for more than one year and causes a variety of problems, such as social impairment, physical discomfort, or mood disorders, that affect daily activities and school performance. Furthermore, TS is often comorbid with other neuropsychiatric disorders, such as attention‐deficit/hyperactivity disorder (ADHD), obsessive‐compulsive disorder (OCD), and mood disorders such as anxiety and depression (Frey and Malaty 2022). However, the etiology and pathogenesis of TS have not yet been elucidated. Most studies suggest that TS may be due to genetic and environmental factors (De et al. 2022; Huang et al. 2022). The cortex‐striato‐thalamocortical loop (CSTC) and neurotransmitter imbalance in the CSTC loop played a vital role in the neurobiological mechanisms of TS (Augustine and Singer 2019). In recent years, there has been increasing acceptance of the immunopathogenesis hypothesis of TS, suggesting that neuroinflammation contributes to the development of TS (Lamothe et al. 2021; Tsetsos et al. 2021). Meryem Ozlem Kutuk et al. examined cytokine levels in children with tic disorders (TD) and found significantly elevated IL‐1β, TNF‐α, IL‐6, and IL‐4, along with lower levels of IL‐17, compared to healthy controls. These findings suggest a pro‐inflammatory state in TD, potentially contributing to its pathogenesis (Kutuk et al. 2024). Genetic factors may contribute to the development of TS by influencing immune system mechanisms. Evidence suggests that specific gene expressions may influence autoimmune mechanisms in patients with TS. Specifically, the expression of genes related to catecholamines, such as dopamine receptors and brain‐derived neurotrophic factor (BDNF), has been associated with inflammatory processes in TS (Marazziti et al. 2023). However, because cerebrospinal fluid (CSF) examination is not mandatory for the diagnosis of TS, reports of neuroinflammation in TS are minimal. In this study, we reported four patients with TS who suddenly had worsening tics and underwent CSF examination to look for possible causes, the results of which did not reveal evidence of intracranial infection or autoimmune encephalitis, but provided clues to the presence of neuroinflammation in TS.

### 1.1 Methods

We retrospectively report on four patients (aged 10–14 years) with TS who were admitted to our hospital between 2019 and 2022 due to a sudden and severe exacerbation of their tics. To investigate possible underlying causes and rule out intracranial conditions such as intracranial infection or autoimmune encephalitis, all patients underwent CSF tests. Written informed consent was obtained from both patients and their parents/guardians prior to these investigations.

### 1.2 Data Collection and Diagnosis

The children were seen by pediatric neurologists with experience in childhood neurodevelopmental disorders. TS and its comorbidities diagnoses were made following the Diagnostic and Statistical Manual of Mental Disorders, Fifth Edition (DSM‐V). The severity of the symptoms was assessed using the Yale Global Tic Severity Scale (YGTSS).

### 1.3 Analysis

Summary statistics were generated for categorical variables. General background information included sex and age, age at the tic episode, severity of the tic, comorbidities, test results, and treatment.

## 2 Case Presentation

In all four cases, routine and biochemistry tests in the CSF, as well as tests for oligoclonal bands and 14 types of neural autoantibodies (anti‐DRD2 antibody, anti‐glutamate receptor antibody (NMDA type, AMPA type 1, AMPA type 2) antibody, anti‐LGI‐1 antibody, anti‐CASPR2 antibody, anti‐GABAB antibody, anti‐IgLON5 antibody, anti‐DPPX antibody, anti‐GlyR1 antibody, anti‐GAD65 antibody, anti‐mGLuR5 antibody, anti‐mGluR1 antibody, anti‐Neurexin‐3α antibody), were negative. The analysis of CSF cytokines revealed significantly elevated levels of MCP‐1 in all patients: 877.6 pg/mL (Case 1), 840 pg/mL (Case 2), 413.3 pg/mL (Case 3), and 435.1 pg/mL (Case 4), compared to the reference range of 0–127 pg/mL. Other cytokines (MIP‐1α, SDF‐1α, IL‐27, IL‐1β, IL‐2, IL‐4, IL‐5, IP‐10, IL‐6, IL‐7, IL‐8, IL‐10, Eotaxin, IL‐12p70, IL‐13, IL‐17A, IL‐31, IL‐1 RA, RANTES, IFN‐γ, GM‐CSF, TNF‐α, MIP‐1β, IFN‐α, IL‐9, TNF‐β, GRO‐α, IL‐1α, IL‐23, IL‐15, IL‐18, IL‐21, IL‐22) were negative in all patients.

### 2.1 Case 1

Case 1 is a 10‐year‐old boy with an onset of multiple motor and vocal tics at age 8. At eight years old, he had episodes of involuntary blinking and vocal utterances. No trigger was found. He was administered aripiprazole for six months, and the symptoms disappeared for a few months, then the child discontinued the medication. However, four months ago, symptoms reoccurred, and one month ago, symptoms were becoming more and more severe. The child twitches almost every hour, seriously affecting his daily life. Although he retook aripiprazole, the effect was minimal, so he came to our hospital for further treatment. The child's birth status, growth and development, and family history were not special. There was no special physical examination on admission. Laboratory tests showed that whole blood count, biochemistry, antistreptolysin O(ASO), C‐reactive protein (CRP), and ceruloplasmin were normal. Due to his sudden worsening tics, the CSF was tested to rule out the possibility of intracranial infection or autoimmune encephalitis. The routine CSF and biochemistry were normal. The CSF was negative for oligoclonal bands and 14 types of neural autoantibodies, and the examination of the CSF cytokine suggested that MCP‐1 was significantly elevated at 877.6 pg/mL. No significant abnormalities were observed on the cranial magnetic resonance imaging (MRI) and electroencephalogram (EEG). YGTSS scores: 21 points for movement, 12 points for vocalization, and 40 points for the degree of functional impairment. The total score was 73 points, suggesting severe. Swanson, Nolan, and Pelham IV Rating Scale (SNAP‐IV) scores: Attention deficit (16 points), hyperactive impulsivity (10 points), and other (19 points) showed that he has comorbid ADHD. Clonidine transdermal patch was added to treat his tics and control ADHD. After a month, his symptoms improved.

### 2.2 Case 2

A 12‐year‐old boy with TS was admitted to our hospital for vocal and motor tics for a duration of five years. He was once treated with aripiprazole and his symptoms improved, so he stopped the medication. At the time of admission, he did not take any medicine for his tics, although he still had symptoms of tics, manifested by twisting the upper body to the left side and clearing his throat. His symptoms worsened a week before admitting to our hospital; it happened much more frequently than before, and worse was that he had a sleep problem; it was hard for him to fall asleep, slept only two to three hours a day, and he became irritable. The child was born full‐term and had a typical growth history. There was no family history of TD. There was no special physical examination at admission. Laboratory tests showed that the whole blood count, biochemistry, antistreptolysin O(ASO); CRP, ceruloplasmin, immunoglobulins (IgG, IgM, IgA, C3, C4), and serum cytokines (IL‐2, IL‐4, IL‐6) were normal. Due to his sudden worsening tics and change of temperament, autoimmune encephalitis was suspected and CSF was tested. Routine CSF and biochemistry were normal. The CSF was negative for oligoclonal bands and 14 types of neural autoantibodies. The examination of CSF cytokines suggested that MCP‐1 was significantly elevated at 840 pg/mL. No significant abnormalities were observed on cranial MRI and EEG. YGTSS scores: 25 points for movement, 21 points for vocalization, and 30 points for degree of functional impairment; the total score was 76, suggesting severe. The child was treated with aripiprazole and, after a week, the child's tic symptoms and irritability were reduced and there was no more disturbance in sleep.

### 2.3 Case 3

The patient, a 13‐year‐old boy, had suffered from tics for eight years. His tic symptoms included blinking, shaking of the head, shaking of the shoulders, and clearing of the throat. The tic form is variable; it can be a single or multiple tics fused into a series of actions. He had been sequencely treated with aripiprazole, tiapride, topiramate, and haloperidol. During the treatment period, the frequency of tics decreased, but symptoms never fully resolved. Recently, his tics worsened and he was less responsive and in a low mood, so he was admitted to our hospital. The child was born by cesarean section at full term, with a typical growth and developmental history and no family history of TD. He was a student in junior high school, but due to TS, he was suspended for one year. After admission, blood routine, biochemistry, CRP, and ASO tests were normal and to exclude autoimmune encephalitis, CSF was tested. The results showed that the CSF routine and biochemistry were normal, and the CSF oligoclonal bands and 14 types of neural autoantibodies were both negative. However, the CSF cytokine test found that MCP‐1 was elevated at 413.3 pg/ml. Cranial MRI and EEG were normal. Depression self‐rating scale: 76.25 points, indicating severe depression; anxiety self‐rating scale: 52.5 points, indicating mild anxiety; YGTSS scores: 20 points for movement, 12 points for vocalization, and 50 points for degree of functional impairment, the total score was 82 points, suggesting severe. His slow response and low mood were considered to be related to his depressive disorder, and he was referred to a psychiatric specialist for treatment.

### 2.4 Case 4

The patient, a 14‐year‐old boy, had suffered from recurrent involuntary tics for nine years and had been aggravated for two months. Nine years ago, he developed involuntary blinking without apparent cause, about 5–6 times a day, with an increase in the frequency of blinking, as well as shoulder shrugging and throat clearing, and was treated with haloperidol, clonidine patch, tiapride, and aripiprazole. One year ago, he developed inattention and hyperactivity, was diagnosed with ADHD, and was treated with atomoxetine. At the time of admission, he was treated with aripiprazole, topiramate, tiapride, and atomoxetine and his motor tics disappeared but he still had vocal tics. In the past two months, the frequency of vocal tics increased and he was significantly more irritable than before. The child was born by cesarean section at full term and with a typical development history. There was no family history of TD. Physical examination did not show any significant abnormalities. Auxiliary examination: blood routine, biochemistry, ceruloplasmin, and IgE were normal. Due to his sudden worsening tics and a change in temperament, a CSF test was ordered to exclude autoimmune encephalitis. The results showed that the CSF routine test, biochemistry, oligoclonal bands, and neural autoantibodies were negative. The CSF cytokine test found that MCP‐1 was elevated at 435.1 pg/mL. No significant abnormalities were observed on cranial MRI. The EEG showed some spikes in the bilateral frontal, right central, and anterior temporal regions during sleep. YGTSS scores: 15 points for vocalization, 40 points for functional impairment, and the total score were 55 points; SNAP‐IV scores: attention deficit (15 points), hyperactive impulsivity (8 points), other (11 points), self‐assessment scale for anxiety and depression: negative. The child was continued with aripiprazole, tiapride, topiramate, and atomoxetine and behavioral interventions were suggested. One month later, the child's tic symptoms improved a little, but his temper improved considerably.

The clinical characteristics of the four cases and the CSF analysis are summarized in Table 1.

**TABLE 1:** Clinical characteristics of the four cases.

Clinical features	Case1	Case 2	Case 3	Case 4
Gender	male	male	male	male
Age(year)	10	12	13	14
Age of onset of tic disorder	8	7	5	5
ASO	—	—	—	—
CRP	—	—	—	—
CSF test				
routine xamination	—	—	—	—
Biochemistry	—	—	—	—
Oligoclonal bands	—	—	—	—
Neural autoantibodies	—	—	—	—
MCP‐1(pg/mL)	877.6↑	840	413.3↑	435.1↑
EEG	Normal	Normal	Normal	During sleep, some spikes appeared in the bilateral frontal, right central, and anterior temporal regions.
MRI	Normal	Normal	Normal	Normal
YGTSS scores				
Moto tics	21	25	20	0
Vocal tics	12	21	12	15
Functional impairment	40	30	50	40
Total	73	76	82	55
Comorbidity	ADHD	Sleep problem	depression	ADHD

Abbreviations: ADHD, attention deficit/hyperactivity disorder; ASO, antistreptolysin O; CRP, C‐reactive protein; CSF, cerebrospinal fluid; EEG, electroencephalogram; MRI, magnetic resonance imaging; YGTSS, Yale Global Tic Severity Scale; −, Negative.

## 3 Discussion

We report the results of the CSF examination in four cases of TS. All four patients were male adolescents and some of them had received multiple medications. However, their symptoms did not resolve and they had comorbidities due to sudden worsening of tics and temperament changes during the disease; intracranial infection or autoimmune encephalitis was suspected as a possibility and an immunological examination of the CSF was performed. The results indicated that the routine and biochemical parameters of the CSF were normal, the oligoclonal bands were negative and 14 types of neural autoantibodies were negative. Among the 34 cytokines tested, only MCP‐1 was significantly elevated. We first found that MCP‐1 was evaluated in the CSF of patients with TS. It was suggested that MCP‐1‐associated neuroinflammation might be the causative mechanism of TS, and treatment targeting MCP‐1 might be able to effectively control symptoms of TS.

To date, only three studies have reported CSF neuroinflammation in TS patients (Claudia Wenzel UWKRMV 2011; Baumgaertel et al. 2019; Pranzatelli et al. 2017); Wenzel et al. (Claudia Wenzel UWKRMV 2011) found positive CSF oligoclonal bands in 8 of 21 patients with TS of different ages; Charlotte et al. (Baumgaertel et al. 2019) found positive CSF bands in 4 of 20 adult patients with TS, with a positivity rate of 20%, which is higher than the 5% positivity rate in the average population. In our study, the four patients had tested the oligoclonal bands, which were negative, consistent with previous studies that only some patients with TS showed positive CSF oligoclonal bands. Another case‐control study explored the cytokine/chemokine profile in TS, including five patients with TS and 26 pediatric neurological patients with non‐inflammatory infections as control. They found that the frequency of subsets of B and T cells or NK cells, the proportion of intracellularly stained T helper type 1 (IFN‐c) or type 2 (IL‐4) cells; the concentrations of chemotactic agents of B cells CXCL13, CXCL10 concentrations; and the proliferation/survival cytokines of B cells BAFF and APRIL, or other chemokines (CCL19, CCL21, CCL22) did not differ significantly in both groups (Pranzatelli et al. 2017). However, the study did not detect the level of MCP‐1. Our four patients had tested 34 items of cytokines/chemokines in the CSF, and only MCP‐1 was found to be elevated, which was consistent with previous studies that found that MCP‐1 gene expression was significantly elevated in the basal ganglia region of patients with TS (Morer et al. 2010). It was suggested that MCP‐1 may play an essential role in the etiology and pathogenesis of TS and may be used as a biological marker.

MCP‐1 plays a crucial role in inflammation and is involved in the pathogenesis of various diseases through various mechanisms.MCP‐1 binds to its CCR2 receptor, activating signaling pathways that regulate cell migration. MCP‐1 can play a crucial role in leukocyte migration across the blood‐brain barrier and promote neuroinflammatory responses in the brain (Cédile et al. 2017). MCP‐1 is lowly expressed in non‐inflammatory states of the central nervous system (CNS) and is highly expressed during inflammation.MCP‐1 is crucial in many CNS diseases, especially those involving inflammation and chronic inflammation in neurodegenerative diseases (Subhramanyam et al. 2019). MCP‐1 plays a role in the pathogenesis and progression of intractable epilepsy, and its expression is upregulated in neurons and glial cells in the epileptogenic zone (Yamanaka et al. 2021); the more pronounced the frequency of seizures, the significantly higher the expression of MCP‐1 (Cerri et al. 2016). Furthermore, MCP‐1 is significantly elevated in the CSF of patients with ASD (Peng et al. 2021). However, the relationship between MCP‐1 and many neurological diseases has been reported. The relationship between MCP‐1 and TS was not an issue of interest for the researchers. For the first time, we report that MCP‐1 is correlated with TS. Our study found that MCP‐1 was significantly elevated in the CSF of patients with TS, but the exact mechanism is unclear. MCP‐1 has been found to regulate the release of neuronal glutamatergic neurotransmitters (Chen et al. 2020), and long‐term exposure to MCP‐1 could activate the nigrostriatal dopamine system and that unilateral intracerebral injection of MCP‐1 (50 ng) in rats increases extracellular concentrations of dopamine and its metabolites, and in addition, these injections of MCP‐1 lead to increased locomotor activity and rotational behavior, suggesting that MCP‐1 may increase dopaminergic neuronal excitability, dopamine release, and associated locomotor activity (Guyon et al. 2009). The effect of MCP‐1 on glutamatergic and dopamine neurotransmission may also be one of the pathogenic mechanisms involved in TS, which needs to be further confirmed in future studies.

MCP‐1 has a potential prognostic and diagnostic marker value in many diseases. The involvement of the MCP‐1/CCL2‐CCR2 axis in the pathogenesis of various diseases has been demonstrated in several studies, and the clinical targeting of MCP‐1 may be effective in the treatment of inflammation‐related diseases (Singh etal. 2021). Interventions targeting MCP‐1 may help alleviate symptoms of TS, MCP‐1 may be a biological marker of TS, and interventions targeting MCP‐1 may be a new target for the treatment of TS.

Although, the study found that serum anti‐DRD2 antibodies were significantly associated with tic exacerbations in chronic TD (Addabbo et al. 2020), the anti‐DRD2 antibody in the CSF of patients with TS we tested was negative, indicating that the relationship between the anti‐DRD2 antibody and TS needed further research. We also tested some other neural autoantibodies, such as anti‐glutamate receptor antibodies (NMDA type, AMPA type 1, AMPA type 2) antibodies, anti‐LGI‐1 antibody, anti‐CASPR2 antibody, anti‐GABAB antibody, anti‐IgLON5 antibody, anti‐DPPX antibody, Anti‐GlyR1 antibody, anti‐GAD65 antibody, anti‐mGLuR5 antibody, anti‐mGluR1 antibody, anti‐Neurexin‐3α antibody, all were negative, and this was the first report of these neural autoantibodies in the CSF of patients with TS.

In this study, we observed a significant increase in MCP‐1 levels in CSF of children with TS during periods of exacerbation of symptoms. This finding is particularly noteworthy, as MCP‐1 is known to play a critical role in neuroinflammation and immune cell recruitment, indicating that it may be involved in the inflammatory processes that underlie TS. The elevated levels of MCP‐1 observed in our patients suggest that immune dysregulation may be related to the pathophysiology of TS, which is consistent with the increasing evidence supporting the role of neuroinflammation in TD.

However, several limitations must be considered. The retrospective nature of this study, coupled with the absence of a control group, limited our ability to draw clear conclusions about the specificity of MCP‐1 elevation to TS. Prospective studies with appropriate implications in the future are crucial to validate our findings and strengthen the study design. Furthermore, the lack of baseline and follow‐up data limits our ability to link fluctuations in MCP‐1 levels to the timing and severity of TS exacerbation. Without longitudinal data, we cannot determine whether elevated MCP‐1 is a cause or consequence of the worsening symptom. This highlights the need for future longitudinal design studies that can evaluate MCP‐1 levels at multiple time points. These studies will help establish whether changes in MCP‐1 correlate with symptom exacerbations or reflect the underlying inflammatory state, and whether MCP‐1 could serve as a biomarker for disease progression or treatment response in TS.

## 4 Conclusions

In this section, we present the results of the CSF of four patients with TS, and for the first time we found that MCP‐1 was significantly elevated in the CSF of TS patients, suggesting that MCP‐1‐related neuroinflammation may be involved in the pathogenic mechanism of TS. MCP‐1 may be used as a biomarker and a new target for the diagnosis and treatment of TS. Further research is needed to validate these findings.

## Author Contributions

**Ke Zhongling**: funding acquisition, writing–original draft, methodology. **Chen Mengxin**: investigation, data curation. **Huang Yuxian**: investigation, data curation. **Chen Yanhui**: conceptualization, writing–review and editing, supervision.

## Ethics Statement

The study was approved by the Ethics Committee of Fujian Medical University Union Hospital.

## Consent

Written informed consent was obtained from the children and their parents to publish this case report.

## Conflicts of Interest

The authors declare no conflicts of interest.

## Peer Review

The peer review history for this article is available at https://publons.com/publon/10.1002/brb3.70617

## Data Availability

All data generated or analyzed during this study are included in this published article.
